# Evolving Bacterial Fitness with an Expanded Genetic Code

**DOI:** 10.1038/s41598-018-21549-w

**Published:** 2018-02-19

**Authors:** Drew S. Tack, Austin C. Cole, Raghav Shroff, Barrett R. Morrow, Andrew D. Ellington

**Affiliations:** 1 National Institute for Standards and Technology, Gaithersburg, Maryland USA; 2 Center for Systems and Synthetic Biology, University of Texas at Austin, Austin, Texas USA

## Abstract

Since the fixation of the genetic code, evolution has largely been confined to 20 proteinogenic amino acids. The development of orthogonal translation systems that allow for the codon-specific incorporation of noncanonical amino acids may provide a means to expand the code, but these translation systems cannot be simply superimposed on cells that have spent billions of years optimizing their genomes with the canonical code. We have therefore carried out directed evolution experiments with an orthogonal translation system that inserts 3-nitro-L-tyrosine across from amber codons, creating a 21 amino acid genetic code in which the amber stop codon ambiguously encodes either 3-nitro-L-tyrosine or stop. The 21 amino acid code is enforced through the inclusion of an addicted, essential gene, a beta-lactamase dependent upon 3-nitro-L-tyrosine incorporation. After 2000 generations of directed evolution, the fitness deficit of the original strain was largely repaired through mutations that limited the toxicity of the noncanonical. While the evolved lineages had not resolved the ambiguous coding of the amber codon, the improvements in fitness allowed new amber codons to populate protein coding sequences.

## Introduction

Since the fixation of the genetic code evolution has been confined to the 20 canonical amino acids, with some incursions by selenocysteine and pyrrolysine. Alternative codon tables (e.g. mitochondrial genomes) are likely evolved from the standard codon table and provide evidence that the canonical genetic code can evolve^1^. A number of theories for the evolution of codon assignment and re-assignment have been proposed^2–4^, and directed evolution experiments have demonstrated the code is not as frozen as once believed^5–7^. However, a full accounting of how a cell might adapt to an expanded genetic code has yet to be presented.

Expanding the standard set of proteinogenic amino acids can be accomplished through changes to the underlying translational machinery. Orthogonal translation systems (OTSs) comprising aminoacyl-tRNA synthetase (aaRS)/suppressor tRNA pairs have been developed that do not significantly interact with the host translational machinery or interfere with already occupied portions of the genetic code^8–10^. Typically, these OTSs allow the incorporation of noncanonical amino acids (ncAAs) by suppressing the amber stop codon (UAG).

Unsurprisingly, cells containing an active OTS often exhibit fitness deficits^11^, possibly because any protein terminated by an amber codon can be unnaturally extended. Efforts to knockout the protein responsible for termination at amber codons, release factor 1 (*prfA*), support this claim: some strains lacking *prfA* were found to be viable only when essential genes terminating with an amber stop codon were recoded to terminate with an alternative stop codon^12^. In order to avoid these fitness impacts upon adopting a new code that would otherwise result in its rejection, previous studies with expanded genetic codes have either relied on bacteriophage, where the fitness of the host organism is irrelevant^7,13^, or have relied on strains that entirely lack amber codons^14^, allowing ready capture of the eliminated codon to create a 21 amino acid genetic code^15^.

Here we utilize an engineered β-lactamase (*bla*) that is structurally dependent on OTS incorporation of the ncAA 3-nitro-L-tyrosine (3nY)^9^. This ‘addicted’ *bla* has allowed us to overcome fitness deficits and carry out long term evolution experiments with an ambiguous amber codon without loss of the underlying OTS. This system models the ‘ambiguous intermediate’ hypothesis of genetic code evolution, which proposes that translation of a specific codon can change by first becoming ambiguously translated before losing ambiguity and gaining specificity for a different amino acid. Here we demonstrate that our system stably incorporates an ncAA for 2,000 generations of evolution, and for the first time identify the entire complement of genomic mutations that lead to improved fitness in the presence of an enforced 21 amino acid code.

## Results

### Experimental set-up

**Figure 1:**
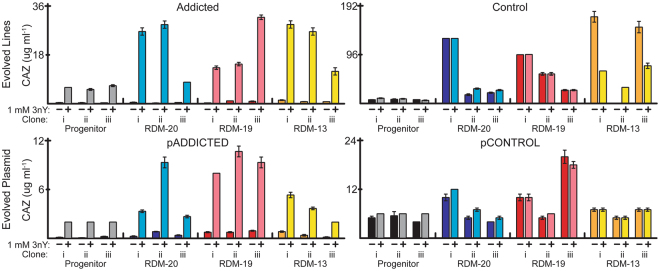
Ceftazidime MICs. MICs of progenitor cells (gray and black) and evolved lines in the absence and presence of 1 mM 3nY. All lines increased MICs during evolution (upper panels, compare gray/black to colored bars). Lines addicted to 3nY remained dependent on 3nY for ceftazidime resistance after 2000 generations (upper left) while control lines never required 3nY for ceftazidime resistance (upper right). Plasmids extracted from evolved lines and transferred to wild-type *E*. *coli* strain MG1655 showed smaller increases in ceftazidime resistance (lower graphs). Values are the average of biological triplicates, error bars represent s.e.m.

We wished to examine the long-term adaptation and evolution of *E*. *coli* addicted to a ncAA, 3nY. We assembled an OTS for the incorporation of 3nY comprised of a *Methanocaldococcus jannaschii* tyrosyl-aaRS variant that had previously been engineered to be specific for 3-iodo-L-tyrosine^10^ but was also compatible with 3nY^16^, and the corresponding *M*. *jannaschii* tyrosyl-tRNA in which the anticodon was complementary to the UAG amber stop codon. This OTS enabled ‘addiction’ via a β-lactamase variant (*bla*_TEM-1.B9_) that had been previously selected to be dependent upon 3nY incorporation at amino acid position 162^16^. However, since *bla*_TEM-1.B9_ with 3nY already conferred resistance to high levels of ampicillin we further engineered *bla*_TEM-1.B9_ to use ceftazidime (CAZ) as a substrate^17,18^. The new β-lactamase, *bla*_Addicted_, conferred moderate resistance to CAZ in a 3nY-dependent manner at concentrations commonly used in bacterial cultures, with a measured minimal inhibitory concentration (MIC) of ceftazidime of 3–10 μg mL^−1^ (see Fig. 1). This lower MIC allowed us to both retain and challenge the 3nY incorporation by progressively increasing CAZ concentrations during culture.

The OTS and *bla*_Addicted_ were assembled together on a plasmid (pADDICTED). We also constructed a control plasmid (pCONTROL) by replacing the 3nY codon (UAG) at position 162 in *bla*_Addicted_ with a phenylalanine codon (UUU), generating *bla*_Control_. Phenylalanine is the only canonical amino acid that produces a functional β-lactamase when replacing 3nY162 in *bla*_TEM-1.B9_^16^. As expected, pCONTROL conferred CAZ resistance in a 3nY-independent manner (Fig. 1).

**Figure 2:**
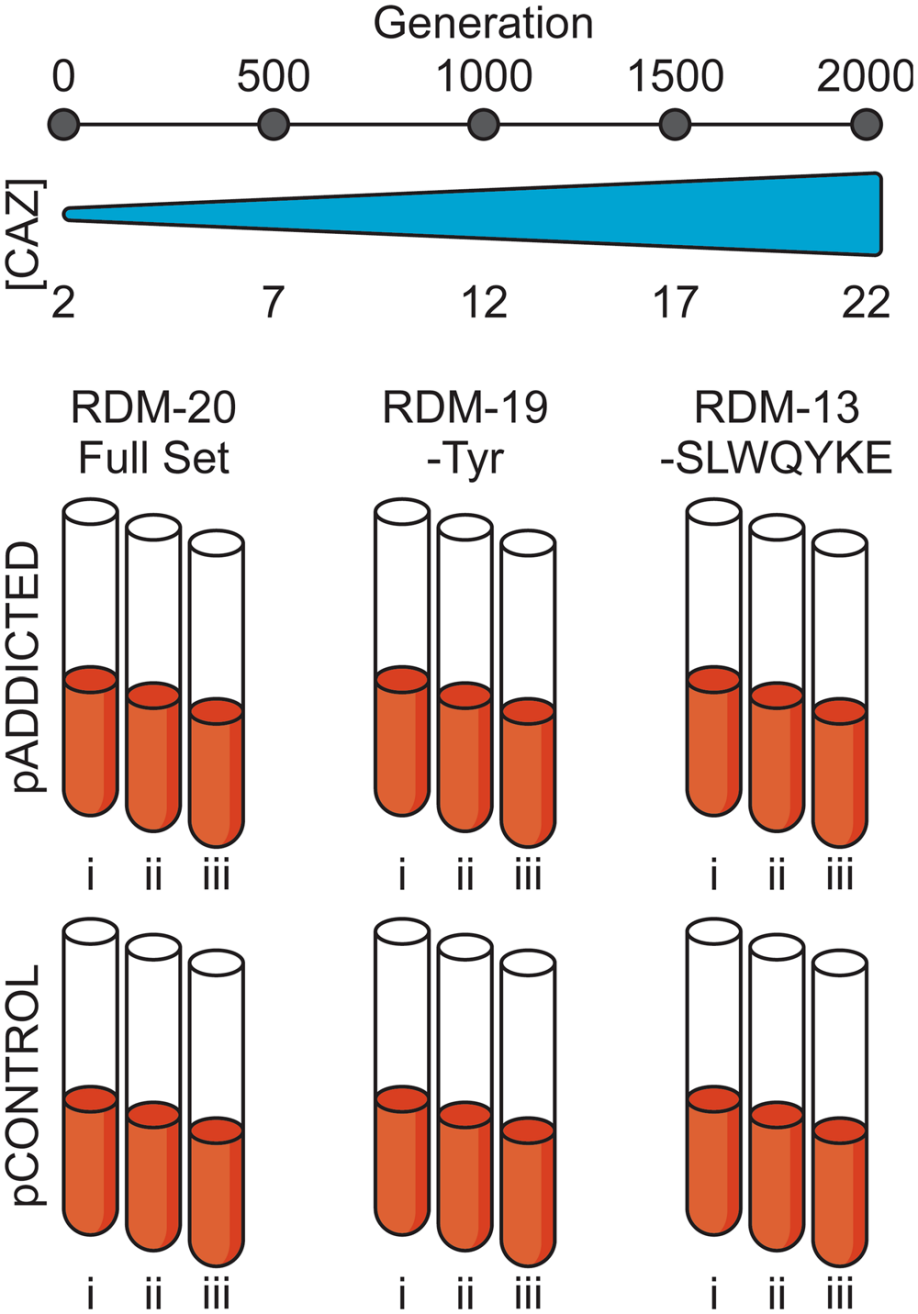
Evolution set up. Wild-type *E*. *coli* strain MG1655 containing either pADDICTED or pCONTROL was evolved in biological triplicates (i, ii, and iii) for 2000 generations in one of three rich defined media conditions each supplemented with 10 mM 3nY. The first (RDM-20) contained all 20 canonical amino acids, the second (RDM-19) lacked tyrosine, and the third (RDM-13) lacked seven amino acids (serine, leucine, tryptophan, glutamine, tyrosine, lysine, and glutamate). During evolution ceftazidime concentration was increased to provide a fitness burden and enforce OTS activity.

As a chassis for evolution, we chose to use *E*. *coli* strain MG1655 because it is well-characterized, with a sequenced and annotated genome^19^. MG1655 is autotrophic for all 20 canonical amino acids allowing for robust growth in amino acid knockout media. MG1655 was transformed with pADDICTED or pCONTROL, and lines were passaged in three different mixtures of amino acids in MOPS-EZ Rich Defined Medium (RDM). The first mixture contained all 20 standard amino acids (RDM-20), the second mixture lacked tyrosine (RDM-19), and the third mixture lacked seven amino acids; serine, leucine, tryptophan, glutamine, tyrosine, lysine, and glutamate (RDM-13) (Fig. 2). These seven amino acids represent all amino acids encoded by codons accessible through single nucleotide mutations from the UAG stop codon; by limiting the charging of the tRNAs for these amino acids, it should prove more difficult for any single mutation in a codon to be readily suppressed by mutations to tRNA anticodons or by mis-pairing. The RDM-13 media condition also proved a more stringent challenge to growth and evolution. Each media condition was supplemented with 10 mM 3nY, matching the concentration of L-serine, the most abundant amino acid in RDM.

### Experimental evolution of bacteria in the presence of 3nY

**Figure 3:**
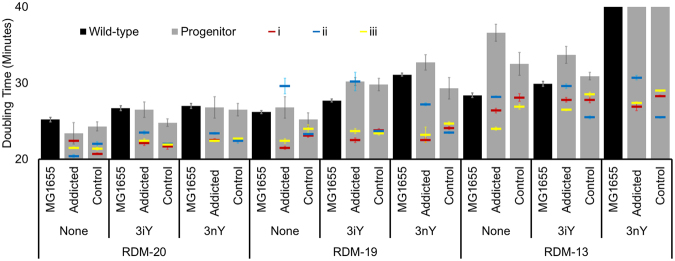
Doubling time (in minutes) of wild-type *E*. *coli* strain MG1655 (black bars), and MG1655 containing the plasmid pADDICTED or pCONTROL (gray bars). Doubling times were measured in all three media conditions (RDM-20, 19, and 13) and with no ncAA (none), with 10 mM 3-iodo-tyrosine (3iY), or with 10 mM 3-nitro-tyrosine (3nY) before evolution (gray bars). Doubling times of each evolved lineage (i, ii, and iii for each condition) were measure after 2000 generations of evolution (red, blue, yellow, respectively) in the media in which they were evolved, without ncAA, with 10 mM 3iY, or with 10 mM 3nY. Reported values are based on a minimum of two growth curves from biological replicates, error bars represent s.e.m.

We initiated selections with six independent clones, three containing pADDICTED, and three containing pCONTROL. These lines were denoted as Addicted(i), Addicted(ii), Addicted(iii), and Control(i), Control(ii), Control(iii) (Fig. 2). Each clone was passaged in the three different amino acid environments described above, and evolved lines are identified by the evolutionary media condition and the progenitor clone from which it was initiated (e.g. Addicted-20(i), Addicted-19(i)…Control-13(iii)). Growth cultures were passaged daily by inoculating 5 mL of RDM with 1 µL of overnight growth. This resulted in approximately 12.5 generations per daily passage. While passaging, CAZ concentration was increased at a rate of 1 µg mL^−1^ per 100 generations to a final concentration of 22 µg mL^−1^ to provide evolutionary pressure and ensure enforcement of 3nY dependence. While wild-type MG1655 was capable of growth in all media conditions, the progenitor clones transformed with pADDICTED or pCONTROL proved largely incapable of growth in RDM-13 when supplemented with 3nY, even in the absence of CAZ (Fig. 3, Supplementary Figure 1), so for the first 125 generations (10 passages) in RDM-13 the media was supplemented with 25% RDM-19. After these initial 10 passages, all lines were capable of growth in RDM-13.

At the conclusion of 160 passages, corresponding to approximately 2000 generations, we selected a single clone from each evolved line and sequenced the bacterial genomes of the single clone as well as the entire bacterial culture using the HiSeq. 4000 Illumina platform. Selected cells were, in general, genotypically representative of the average bacterial population. We further characterized phenotypic parameters of the selected evolved cells, as well as the progenitor cells.

### Growth Rates

We used growth rates, evaluated as doubling times, as a general indicator of cellular fitness both before and after evolution. Doubling times were determined for the progenitor cells in all three media conditions, and the doubling time of the evolved lines was calculated in the media in which they were evolved. Doubling time calculations were automated using the software program *GrowthRates*^20^. In general, doubling times were measured in the absence of ceftazidime, and in three different amino acid environments: without ncAA, with 3nY, or with 3iY. Additionally, doubling times were measured with 2 or 22 µg mL^−1^ CAZ (progenitor and evolved cells, respectively), both with and without 3nY.

We first wanted to evaluate the effect amino acid supplements had on *E*. *coli* in the absence of 3nY. We determined the doubling time of wild-type *E*. *coli* strain MG1655 in RDM-20, which compared well to previously reported doubling times of MG1655 in rich media^21^. We found statistically significant increases in the doubling time of MG1655 with the removal of amino acids (compare the doubling times of wild-type MG1655 in RDM-20, RDM-19, and RDM-13, in Fig. 3, p-value calculations in Supplementary Table 1). We then evaluated the effect the OTS had on fitness in the absence of ncAAs and found that doubling times were not significantly affected by the addition of the OTS (compare doubling times of wild-type MG1655 to MG1655 containing pADDICTED or pCONTROL, within RDM-20 or RDM-19). Somewhat surprisingly, the addition of the OTS in RDM-13 did show a statistically significant increase in doubling time when encoded on pADDICTED, but not pCONTROL. This increase in doubling time may be a fitness effect due to *bla* truncation at the UAG codon of *bla*_Addicted_, as doubling times of MG1655 with pADDICTED are generally higher than MG1655 with pCONTROL in all conditions, despite identical plasmid sequence except for the single amber codon in the *bla* gene (Fig. 3, compare pADDICTED and pCONTROL before evolution in each RDM/ncAA combination). Together, these results suggest that the amino acid environment directly affects growth rates, and that encoding of the OTS on a plasmid on its own does not have a profound effect on fitness.

We next wanted to evaluate the effect ncAAs had on *E*. *coli* fitness. We evaluated wild-type MG1655 in RDM-20, -19, and -13 with 10 mM 3iY or 10 mM 3nY and found significant increases in doubling times in all three conditions when compared to media without ncAAs (Fig. 3). The addition of an OTS did not significantly increase doubling times of MG1655 in ncAA enriched RDM-20 or RDM-19 when compared to the doubling time of wild-type MG1655 in the same media. However, in RDM-13 the addition of 3nY significantly increased the doubling time of wild-type MG1655, and the addition of the OTS further exacerbated this fitness challenge, preventing growth entirely. Surprisingly, the addition of 3iY to RDM-13 did not have a significant effect, with doubling times similar to growth in RDM-13 without ncAA. These results suggest 3nY is mildly toxic and 3iY less so, but the fitness burden is significantly enhanced in minimal amino acid environments (RDM-13), particularly when the OTS is encoded and suppression is possible. These latter results are consistent with our findings in the absence of ncAAs.

Over the course of evolution, the doubling times of all lines decreased in all conditions (Fig. 3), indicating that minimal media itself is a fitness burden for the strains, including the parental strain, and thus that many of the genomic mutations we observed (below) were generally improving the fitness of the strains for supplemented minimal media. The relative fitness effects associated with media conditions remained consistent even after evolution; doubling times were fastest in RDM-20, and slowed with greater metabolic stresses with decreasing numbers of amino acid supplements. Surprisingly, the large growth deficits in RDM-13 were largely overcome during evolution, with evolved cells reaching doubling times near that of wild-type MG1655 grown in richer conditions (i.e., compare with RDM-20 without ncAA to doubling time of evolved lines in RDM-13 with 3nY in Fig. 3).

There were two exceptions to these general observations: Control-13(ii), which showed no growth in media lacking ncAA at the conclusion of the experiment (Fig. 3, Supplementary Figure 1), and Addicted-19(ii), which showed reduced growth (increased doubling time) in the absence of 3nY, even when supplemented with the nominally less toxic 3iY.

### Evolution of antibiotic resistance

During the course of passaging, CAZ concentrations were increased to levels beyond the MIC of the progenitor cells (the initial MIC of lines was approximately 2–6 µg mL^−1^, while the final CAZ concentration challenge was 22 µg mL^−1^). Bacterial survival at increasingly higher CAZ concentrations indicated that CAZ resistance was evolving in the lines. We measured the MIC of each evolved clone with and without 3nY (Fig. 1). The addicted lines remained dependent on 3nY for improved ceftazidime resistance, while the MICs of control lines increased in a 3nY-independent manner.

All nine of the addicted cell lines acquired at least a single mutation in *bla*_Addicted_ during evolution (Table 1). Several of these mutations are known or expected to be stabilizing mutations, while others are known to expand substrate specificity of *bla*_TEM-1_^22–27^. Other mutations are specific to *bla*_TEM-1.B9_, which originally included a number of substitutions relative to the wild-type *bla*_TEM-1_ that addicted it to 3nY. The substitution T139I in Addicted-20(iii) and Control-19(ii), is more similar to the original wild-type residue, leucine, but does not reduce the 3nY dependence of the enzyme. In contrast, only five control lines acquired mutations in *bla*_Control_: Control-20(i), -20(ii), -20(iii), -19(i), and -19(iii). The higher rate of mutation for *bla*_Addicted_ relative to *bla*_Control_ (p = 0.0054) indicates that the enzyme dependent upon the ncAA was not initially as fit as its non-addicted counterpart, especially in the presence of increasing antibiotic concentrations.

**Table 1:** Mutations found in *bla*_Addicted_ and *bla*_Control_ in evolved lines.

Mutation	Lines	Effect	Previously Identified
V33I	Addicted-13(ii)	—	^22,58^
Q39K	Control-20(ii)	Expanded Substrate Activity	^27^
G92D	Addicted-20(i), -20(ii), -19(ii)	Stabilizing	^22,59^
T139I	Addicted-20(iii), Control-19(ii)	*bla*_TEM-1.B9_ Specific	—
T140K	Addicted-19(ii), -19(iii), -13(iii)	—	—
M152I	Control-20(i)	*bla*_TEM-1.B9_ Specific	—
H153R	Addicted-20(ii)	Stabilizing	^60,61^
M155I	Control-20(iii)	—	^22,62,63^
M182T	Addicted-19(i)	Stabilizing	^25–27^
A184V	Addicted-13(i)	Expanded Substrate Activity	^64,65^
T200P	Addicted-20(i)	—	—
A224V	Control-19(i)	Stabilizing	^25^

The phenotypic impact of the *bla* mutations was further examined by purifying the plasmids pADDICTED and pCONTROL from the evolved lines and transforming them into wild-type MG1655. These evolved plasmids generally conferred higher MICs than the progenitor plasmids (Fig. 1). Notably, pADDICTED from lines Addicted-20(ii), -19(i), -19(ii), -19(iii), and -13(i) yielded MIC increases greater than 2-fold the original MIC. These plasmids all contained *bla* mutations known or suggested to have stabilizing effects (Table 1). However, *bla* mutations cannot fully explain MIC changes, as the original lines are generally more resistant to CAZ than are wild-type MG1655 cells with the evolved plasmids transformed (compare upper panels of Fig. 1 with lower panels). Moreover, *bla*_Addicted_ in lines Addicted-19(iii) and Addicted-13(iii) each have only a single, identical mutation (T138K) yet confer different MICs when transformed into wild-type *E*. *coli* strain MG1655.

Beyond mutations in the *bla* gene itself, increases in MIC may have also been the result of other plasmid or genomic mutations that altered antibiotic resistance through other mechanisms. For example, the plasmid from Addicted-13(iii) has a mutation in *repC*, which can affect copy number and in turn antibiotic resistance^28,29^. In addition, genomic mutations occurred in genes that are known to have a role in antibiotic tolerance. Four lines (Addicted-20(i), -20(iii), -19(iii), and Control-20(iii)) had mutations in *envZ* (Table 2), a histidine kinase that regulates *ompF* and *ompC* expression, which in turn alter membrane porosity and have been tied to β-lactam resistance^30,31^. In the same vein, Addicted-13(iii) has a mutation directly in *ompF*. Mutations to *cyaA* or *crp*, two related proteins directly or indirectly involved in transcriptional regulation, occurred in eight evolved lines (Addicted-20(ii), -19(ii), -19(iii), -13(i), -13(iii) and Control-19(i), -19(iii), -13(iii)), and inactivation of these genes has been shown to produce resistance to β-lactams^32,33^. The *opgG* gene was mutated in Addicted-20(iii), -19(i), and Control-20(ii), and is involved in conferring resistance to antibiotics^34^. Finally, lipopolysaccharide (LPS) expression has been tied ceftazidime treatments, especially at or near MIC levels, and several lines had mutations in LPS biosynthesis and maintenance genes. Notably, seven lines had IS insertions in the *waa* operon that encodes the core oligosaccharide of LPS, including *waaO* (Addicted-20(ii), -19(i), and Control-20(ii), -20(iii)), *waaQ* (Control-20(i)), *waaP* (Addicted-13(i)), and *waaB* (Control-19(ii)). Also, three lines had mutations in the LPS-related gene *galU*, (Control-19(i), -13(i), -13(iii)), and one line had a mutation in *lptD*, which is involved in the assembly of LPS at the surface of the cell (Control-13(ii))^35,36^.

**Table 2:** Genomic Mutations of Evolved Lines.

Addicted				Control			
Line	Gene	Mutation	Position	Line	Gene	Mutation	Position
20(i)	sdaC	+9 insert (IS1)	925/1290	20(i)	sdaC	+9 insert (IS1)	1192–1200/1290
	yqiG	A > G	815/2439		waaQ	+9 insert (IS1)	581–589/1035
	envZ	M58R			ecpR/ykgL	C > T	311481
20(ii)	ydeT	A > C	1108/1227	20(ii)	opgG	+9 (IS1)	738–746/1656
	waaO	+9 insert (IS1)	531–539/1020		sdaC	**W18tag**	
	mtdL	A324T			waaO	+4 (IS5)	925–928/1020
	cyaA	−78	2420–2497/2547	20(iii)	sdaC	+1	798/1290
	dnaA/rpmH	−6	3883825		envZ	S87Y	
20(iii)	opgG	**Q314tag**			waaO	+5 (IS2)	100–104/1020
	clsA	+9 (IS1)	1307–1315		yidD	A14G	
	sdaC	A383V		19(i)	ybbP	A580E	
	yghG	G63V			galU	H71L	
	envZ	F107C			uspC-tyrP	−11258 (IS1)	
	ykgM/ykgR	+9 (IS1)	313109		garD	K343Q	
19(i)	opgG	+9 insert (IS1)	385–393/1536		tusD	G43G	
	ycjO	−87	250–336/882		cyaA	D231G	
	waaO	+4 insert (IS5)	533–536/1020	19(ii)	opgH	H417D	
	ycaA	−1	2033/2547		uspC-tyrP	−11067	
19(ii)	yahF	K208R			waaB	+4 (IS5)	226–229/1080
	dosP	I282I			prpB/prpC	A > G	349642
	uspC-yecH	−10333 (IS1)			lacZ/lacI	2 bp > TT	366351
	lhgO	R346W			lacZ/lacI	C > T	366409
	yqeK	L30(tga)			lacI/mhpR	G > A	367573
	crp	F137L			ompF/asnS	G > A	987138
	tauA/tauB	G > T	386203		yhfA/crp	A > C	3485942
19(iii)	hofC	S27R		19(iii)	caiC	I449S	
	uspC-yecA	−12537 (IS1)			quuD	+5 (IS2)	233–237/384
	ppx	−1	365/1542		tyrP	**W357tag**	
	rrlD	C > T	2209/2904		cyaA	D184N	
	envZ	G85V			yihY	R208L	
	dnaA	−54	251–304/1404	13(i)	ftsW	L141I	
	cyaA	R160L			galU	+12 (IS4)	695–706/909
	rrlD	C > T	3424575		uspC-yecA	−11714 (IS1)	
	yihU/yihV	G > T	4073632		tyrA	M117I	
13(i)	flgJ	+9 insert (IS1)	157–165/942		kduI	M193I	
	uspC-tyrP	−10976			mtr	−6	712–717/1245
	rpoD	P97L			malA/yfdC	G > T	2465297
	mtr	−6	712–717/1245		yhfA/crp	+5 bp (IS2)	3486024
	waaP	+4	413–416/798	13(ii)	lptD	**Q557tag**	
	cyaA	D231V			uspC-tyrP	−11031 (IS1)	
13(ii)	pykF	I264T			yhcG	S136A	
	uspC-tyrP	−11298 (IS1)			tnaA	+5 (IS2)	303–304/1416
	sanA	+9 (IS1)	165–173/720		ompF/asnS	+9 (IS1)	987064
	mtr	−6	391–396/1245		topA/cysB	+4 bp (IS4)	1333763
	ompR	M57L			mtr/deaD	T > C	3305872
	yibA	+9 (IS1)	15–23/843	13(iii)	galU	+9 (IS1)	81–89/909
	waaQ/waaA	C-T	3808189		uspC-tyrP	−11280	
13(iii)	yaiT	A > C	682/1458		mtr	L400R	
	ompF	+9 (IS1)	323–331/1089		mtr	−1	1011/1245
	cysB	−1	610/975		cyaA	C120Y	
	uspC-tyrP	−11018 (IS1)			rpsF	D13G	
	mtr	−6	712/717/1245		lrhA/alaA	A > C	2407094
	glpD	+9 (IS1)	1109–1117/1506				
	cyaA	P301L					
	yiiQ	K74taa					

Genomic mutations which occurred after 2000 generations of evolution. Mutations which appeared after 125 generations in RDM-13 are underlined. The four in-frame amber codons which arose in ORFs are bold.

### Retention of the OTS

**Figure 4:**
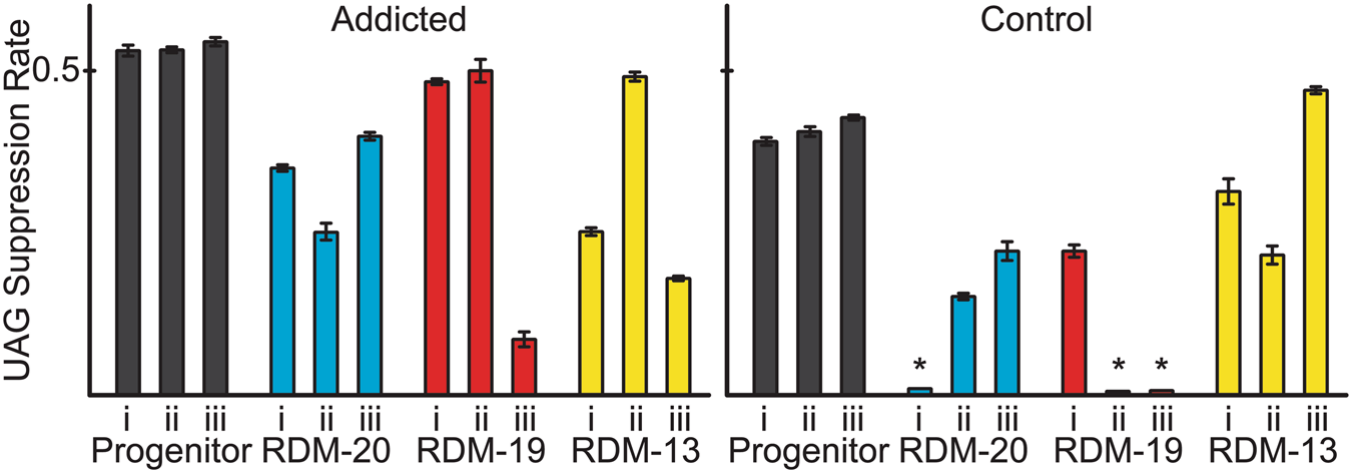
UAG Suppression Efficiency. OTS activity of progenitor cells (black) and evolved lines (colored bars) was determined by measuring UAG suppression efficiency with a GFP reporter. All addicted lines maintained an active OTS (left), while three control lines lost OTS activity over 2000 generations (see ‘*’). Values are the average of four biological replicates, error bars represent s.e.m.

One of the primary questions we were addressing was whether lines that were addicted to the OTS would retain and utilize the ncAA differently or better than lines that were not addicted to the OTS. The 3nY dependent MICs (Fig. 1) strongly suggests that a functional OTS has been retained in all addicted lines. To directly evaluate the functionality of the OTS we measured UAG suppression efficiency with a GFP reporter system that contained a tyrosine (TAT), amber (TAG), or ochre (TAA) codon at position Y39 of GFPmut2^37^. We tested each GFP reporter in progenitor cells as well as each of the evolved lines (Supplementary Figure 2). All of the addicted lines remained capable of efficient amber suppression (>10% 3nY incorporation at UAG codons) (Fig. 4). In contrast, three of the nine control cell lines (Control-20(i), -19(ii), -19(iii)) had lost UAG suppression capability (<1% 3nY incorporation) (Fig. 4, see ‘*’). Surprisingly, UAG suppression efficiency generally dropped in both addicted and control lines relative to their progenitor cells.

**Figure 5:**
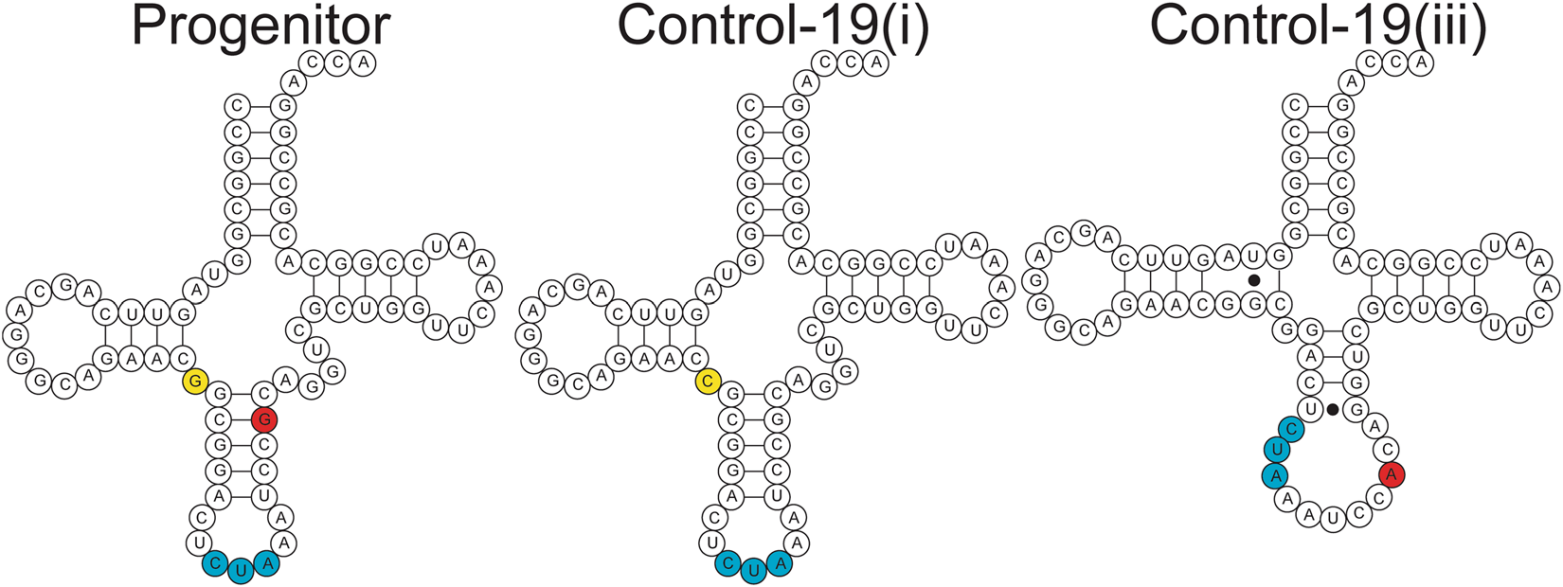
Predicted tRNA structures. Mutations to the OTS-tRNA occurred in two lines during evolution. Computationally predicted tRNA structures from these evolved lines shows substitution G26C (yellow) found in Control-19(i) (center) is not likely to affect general structure or the anticodon (blue), while substitution G42A (red) in line Control-19(iii) is predicted to disrupt tRNA structure (right).

Of the addicted lines, only Addicted-19(i) acquired mutations in the OTS, acquiring a T- > G mutation in the tRNA processing machinery at position T(-28). In contrast, OTS mutations occurred in Control-20(i), -19(i), -19(ii), -19(iii), three of which had lost OTS activity. Of these, lines Control-20(i) and Control-19(ii) have aaRS mutations: Control-20(i) has a frameshift in the aaRS resulting in a UGA ochre stop codon at amino acid position 123, which leads to the complete loss of UAG suppression, and Control-19(ii) contains an IS-mediated insertion in the aaRS, a mechanism that has previously been observed to inactive OTS machinery^11^. Control-19(i) and Control-19(iii) each had mutations in the tRNA. The tRNA of the OTS from Control-19(i) contained a G26C substitution in the hinge between D-stem and anticodon stem (Fig. 5, yellow), while OTS tRNA from Control-19(iii) has a G42A substitution in the anticodon stem of the tRNA (Fig. 5, red). The latter mutation is predicted to compromise tRNA structure, as determined using mfold^38^ and tRNA prediction software^39^ (Fig. 5). This tRNA mutation likely explains the complete loss of UAG suppression in Control-19(iii) (Fig. 4).

Overall, either suppression in general or the specific incorporation of 3nY puts cells at a fitness disadvantage, and in the absence of addiction to the ncAA this trait can be readily lost. The evidence suggests that suppression in general is less toxic than 3nY incorporation. First, in the most restricting environment (RDM-13) the parental strain can take up and incorporate 3iY into proteins, which affects doubling times less than the incorporation of 3nY (Fig. 3, compare doubling times in RDM-13 of wild-type *E*. *coli* strain MG1655 in the presence of 3iY with no plasmid, and with pADDICTED or pCONTROL, each carrying an active OTS for 3iY incorporation). Second, in the absence of addiction the OTS is readily lost or compromised during evolution (Fig. 4, compare suppression efficiencies in ‘Addicted’ and ‘Control’ panels). Additionally, previous work also supports the hypothesis that suppression is not particularly toxic, given the existence and persistence of natural amber suppressors which have minimal effect on fitness or the transcriptome^40^.

### Genomic adaptations

Full genome sequencing and analysis revealed several trends among evolution conditions and the genes affected during evolution. (Table 2). Strikingly, the most commonly mutated or deleted ORFs were amino acid transporters in the hydroxyl and aromatic amino acid permease (HAAAP) family. The most commonly affected HAAAP protein was *tyrP*, a tyrosine-specific permease^41,42^ that was inactivated or deleted in 10 of the evolved lines, including all six evolved in RDM-13, and four of the six lines evolved in RDM-19, including all three control lines, as well as Addicted-19(iii). The most common mechanism (9 of 10 cultures) used to inactivate *tyrP* was an IS1 mediated deletion of a large genomic fragment that excised twelve to fourteen genes, including a portion or the entirety of *tyrP*. The second most commonly mutated HAAAP protein was *mtr*, a tryptophan-specific permease^43^. The *mtr* mutations were found exclusively in lines evolved in RDM-13, with five of the six lines evolved in RDM-13 having a mutated *mtr* gene, and the final line, Control-13(iii), having an intergenic mutation near the *mtr* ORF. The third most common HAAAP protein that was inactivated was *sdaC*, a serine specific transporter, which was inactivated in four of the six lines evolved in RDM-20; a mutation of unknown consequence appeared in a fifth line (A384V). Surprisingly, *sdaC* was unaffected in all RDM-19 and RDM-13 evolved lines.

We hypothesized that deactivation of HAAAPs may have played a role in reducing the fitness burden of 3nY in the media. Evidence for the importance of these mutations for adaptation to growth comes from an examination of the mutations that arose in the RDM-13 lines between generations 0 and 125, which were initially incapable of growth in RDM-13 and had to be supplemented over their first 125 generations (10 passages) with 25% RDM-19 (Fig. 3). We sequenced the genomes of the six RDM-13 lines after 125 generations of growth in the enriched RDM-13 to determine which mutations had occurred early in experimental evolution, and found that five of the six populations had mutations in *mtr* or the *mtr* promoter. These mutations were fixed in three of the five lines, and for the remaining two lines some 22.8% of the Control-13(i) population had a *mtr* mutation, while 44.2% of the Addicted-19(iii) had mutations in *mtr*; both mutations were fixed by generation 2000. Additionally, the *tyrP* region was excised from the genomes of Addicted-13(i) and Control-13(ii). These mutations were the only identifiable mutations in the population after 125 generations, and were likely responsible for the ability to grow in pure RDM-13 after this point. This data supports the hypothesis that aromatic amino acid transporter deactivation (specifically *mtr* and *tyrP*) reduced the fitness burden imposed by the noncanonical aromatic amino acid, 3nY.

### Sampling of new codes

All eighteen lines were initially capable of utilizing 3nY in their proteome, and after 2000 generations of evolution four clones had each acquired a single in-frame amber codon in protein coding sequences. In three of these four instances, the strains did not require 3nY for growth. The clone from Addicted-20(iii) had acquired a SNP resulting in Q314tag in *opgG*, an osmoregulated peptidoglycan biosynthesis protein^44,45^. The other three in-frame amber codons appeared in clones from the Control lines, including Control-20(ii) and Control-19(iii), which acquired a W18tag mutation in *sdaC* and a W357tag mutation in *tyrP*, respectively. These amber substitutions likely down-regulated the expression of these HAAAP genes, consistent with our findings that these loci were frequently deleted or otherwise compromised. Control-19(iii) had deactivated the OTS entirely, indicating W357tag of *tyrP* was a truncation, while in Control-20(ii) the OTS remained partially active, with only ~15% 3nY incorporation (see Fig. 4).

As mentioned above, two lines showed an unexpected dependence on 3nY for growth. When we assayed the ceftazidime MIC for the clone chosen from the Control-13(ii) line there was minimal to no growth on plates without 3nY, but appropriate growth on 3nY supplemented plates. Later, multiple replicates consistently resulted in a no growth phenotype for line Control-13(ii) in the absence of 3nY during amber suppression assays. Sequencing of this genome revealed an in-frame UAG codon in *lptD*, a Q557tag substitution. *lptD* is an essential gene involved in LPS biosynthesis^44,46^. Comparison to the full culture sequencing revealed this mutation was not representative of the bacterial population in the evolving culture Control-13(ii). Despite being a minor component of the full population, this mutation demonstrates the additional mutational space available to the evolving bacteria. An amber stop codon in *lptD* in a wild-type *E*. *coli* would result in a nonviable phenotype, yet with the expanded genetic code this clone has survived.

While the clone from the Control-13(ii) line was found to have an amber stop codon in an essential gene that may explain the lack of growth in the absence of 3nY, we cannot point to a similar rationale for why Addicted-19(ii) shows reduced growth rates in the absence of 3nY. Genome sequencing of the Addicted-19(ii) clone revealed six SNPs had arisen during evolution, four of which caused single amino acid substitutions, a fifth that resulted in a silent mutation, and a sixth that occurred in an intergenic region. As with other strains that had adapted to 3nY toxicity, Addicted-19(ii) contained a 10 kb genomic deletion surrounding the tyrosine permease *tyrP*.

## Discussion

Previously, two different approaches have been used to generate organisms with altered or expanded amino acid genetic codes; a bottom-up approach where components of the organism were engineered to function with a ncAA, or a top-down approach, where an organism was allowed to evolve in the presence of a ncAA^47^. Advances in genome editing and protein structural modeling have made the bottom-up approach feasible, with two recent reports of *E*. *coli* successfully engineered to depend on a ncAA for survival^48,49^. It should now be possible to make fully synthetic genomes^50^ that are designed to have a chemical dependence on an ncAA throughout the genome using the ‘amberless’ *E*. *coli*^51^. Alternatively, top-down approaches have previously been used to generate organisms that preferentially function with a ncAA substituting for tryptophan^5,52^. Using mutagens and selective growth conditions, *Bacillus subtilis* became dependent on a normally toxic tryptophan analog, 4-fluorotryptophan, with only 106 genomic mutations required to change amino acid preference^52^. In contrast, an *E*. *coli* tryptophan auxotroph evolved in the presence of 4-fluorotryptophan in place of tryptophan^53^ could tolerate high levels of the ncAA, but in the end still required tryptophan for growth. A similar approach using long-term evolution in *E*. *coli* allowed for cells to grow using a sulfur-containing tryptophan analog in place of tryptophan^6^. Another recent report shows that the bacteriophage T7 will adopt ncAAs into its genetic code to reach new fitness peaks^7^.

The work described herein can be considered a hybrid approach, resting between bottom-up and top-down. We used a single engineered addiction element, *bla*_Addicted_, to preserve an active OTS over evolutionary timeframes within an otherwise unmodified organism. Thus during 2000 generations of evolution bacteria were required to retain 3nY incorporation as part of its genetic code and could explore the potential utility of 3nY throughout their proteomes. This process simulates the ‘ambiguous intermediate codon’ theory of evolution. Over the described 2000 generations, 3nY-addicted bacteria have remained dependent on 3nY for survival in evolutionary conditions, and cells have evolved to overcome the fitness burdens initially seen from the expanded genetic code. This was not a foregone conclusion: in order to re-establish the canonical 20 amino acid code, lines could have evolved *bla*_Addicted_ to no longer require 3nY, or acquired genomic mutations leading the CAZ resistance without *bla*_Addicted_. Even though 326 genomic amber codons were a single mutational step from stop codons that would not be suppressed by the OTS, in over 2000 generations addicted populations fixed no mutations that led away from amber stop codons used for translational termination, despite previous evidence that recoding amber codons to alternative stop codons reduces the fitness effects of obligate UAG suppression^7^.

Instead it became clear that the most important adaptations to the ambiguous genetic code we had imposed were ones that alleviated either the toxicity of an unnatural amino acid and/or that better optimized the composition of the 20 amino acids normally used for growth. Growth in 3nY clearly led to fitness deficits, and to compensate if the media lacked tyrosine (RDM-19 and RDM-13), the tyrosine permease *tyrP* was inactivated, and if the media also lacked tryptophan (RDM-13), the tryptophan permease *mtr* was inactivated. In contrast, if all 20 amino acids were present (RDM-20), the serine permease *sdaC* was inactivated. Since serine is the most abundant amino acid in RDM conditions (10 mM), it is possible that the amino acid pool is better balanced through deletion of *sdaC*, while still relying on other serine transporters (*sstT*) for serine uptake.

Once strains had evolved to the point where they could accommodate an ambiguous genetic code that could accept suppression with either tyrosine or 3nY, they were positioned for further evolution to specify a new code. After 2000 generations of evolution with 3nY, the existence of three in-frame amber codons in clones with active OTSs, and the fact that a clone from the Control-13(ii) line had adopted an in-frame UAG codon in the essential gene *lptD*, provides evidence that populations are exploring the 3nY mutational space. Additionally, the reduced doubling time of a second line, Addicted-19(ii), in the absence of 3nY may indicate some preference for the new media condition containing 3nY, even though no genomic in-frame amber codons had arisen. In this regard, the evolution of Addicted-19(ii) may resemble the evolution of a *B*. *subtilis* strain that could preferentially utilize 4-fluoro-tryptophan in place of tryptophan^5^.

Overall, our method provides one of the first experiments investigating how a new genetic code is adopted by an organism, and evolved lineages may represent evolutionary intermediates to the adoption of a new amino acid. All lineages overcame the fitness burden associated with 3nY toxicity, but ncAA addiction was required to enforce an active OTS. Further experimentation using the method described here, or similar approaches, will provide insight into the recoding of the genetic code during evolution, and may allow the evolution of biochemically unique organisms.

## Methods

### Evolutionary set up

#### Addiction operon

Plasmid backbone from pMMB67EH^54^ was amplified using DNA oligos (Integrated DNA Technologies) DT01 and DT02 (Supplementary Table 2). The *M*. *jannaschii* OTS and *bla*_TEM-1.b9_ were amplified from a plasmid described previously^16^ using oligos DT03 and DT04. To convert the penicillinase *bla*_TEM-1.B9_ to the cephalosporinase *bla*_Addicted_, residues 165–167 were converted from WEP to YYG using oligo DT05 with either DT06 or DT07 for *bla*_Addicted_ or *bla*_Control_ respectively. Reaction mixtures were transformed into *E*. *coli* TOP10 and selected on LB-agar with 2 μg mL^−1^ CAZ for *bla*_Control_, and the same conditions with 10 mM 3nY for *bla*_Addicted_. Samples were sequence verified at University of Texas core facilities using Sanger sequencing. Properly sequenced plasmid of *bla*_Control_ and *bla*_Addicted_ were used as pCONTROL and pADDICTED respectively, and transformed into *E*. *coli* MG1655. Three colonies from each were selected as clones i, ii, and iii for passaging.

### Media

MOPS-EZ Rich Defined Media (RDM, TEKnova) with the full complement of amino acids (RDM-20), as well as the knockout medias (RDM-19 and RDM-13), were prepared according the manufacturers specification. For media preparation, 3-nitro-L-tyrosine or 3-iodo-L-tyrosine (Sigma-Aldrich) was added to ultrapure deionized water to a final concentration of 17.24 mM. The 3nY or 3iY supplemented water was used to complete RDM, and the entire preparation was filter sterilized with Nalgene Rapid-Flow SFCA filtration units. Prepared media was stored at 4 °C, and moved to room temperature 16–24 hours before use.

### Passaging

Selected colonies i, ii, and iii, from each transformation were picked and grown in RDM-20 supplemented with 10 mM 3nY and 2 μg mL^−1^ CAZ for 16 hours. Each culture was then used to inoculate three subcultures of RDM-20, RDM-19, and RDM13. Initially, cultures were incapable of growth in RDM-13, but were capable of growth when 25% of media was replaced with RDM-19. This supplemented RDM-13 was used for the first 125 generations for RDM-13 evolved cultures, after which cultures were capable of growth in RDM-13. Cultures were passaged every 16–24 hours by transferring 1 μL of culture into 5 mL of fresh media, and grown shaking at 37 °C. A 500 μL sample from each line was preserved at generation 0, 125, 250, and every 250 generations for the duration of evolution, samples are stored in 25% glycerol at −80 °C.

### Genome Sequencing and assembly

After the 2000 generations, 1 μL from each line was streaked onto RDM-agar supplemented with 10 mM 3nY. Two single colonies from each line were selected and grown in 3 mL RDM-20 with 10 mM 3nY with 22 μg mL^−1^ CAZ. Simultaneously, samples from the progenitor cells were grown in similar conditions using 2 μg mL^−1^ CAZ. After overnight growth, glycerol stocks were made of clonal cultures, and genomic DNA was isolated from one of the two cultures using bacteria genome miniprep kit (Sigma-Aldrich). Genomic DNA from mixed cultures were also prepped. Genomes were sequenced using the HiSeq. 4000 platform, 150 bp paired ends, achieving greater than 100× coverage across all samples. Raw reads were processed through trimming and adapter removal using trimmomatic (v0.32)^55^. Alignment of sequencing reads and variant calling was performed through the breseq workflow (v0.27.2)^56^.

### Growth Curves

Glycerol stocks were used to inoculate 2 mL of appropriate RDM and incubated overnight while shaking at 37 °C. Then, 1 µL from overnight cultures were used to inoculate 100 µL of media with appropriate concentrations of CAZ and 3nY. A Tecan Infinite M200 pro microplate reader set to 37 °C monitored absorbance at 600 nm. Data was processed by subtracting background on per well basis and trimming data from early- to mid-log phase (0.05 to 0.2 OD) for optimal cellular concentration estimations^57^. Doubling times were then calculated using GrowthRates program^20^. All reported growth rates and based on duplicate growth curves (two or more), with multiple internal measurements taken from within each curve, as described^20^, and reported with standard error.

### MICs

Progenitor and evolved cells were grown overnight in 3nY enriched RDM with 2 μg mL^−1^ or 22 μg mL^−1^ of CAZ respectively. Overnight cultures were used to inoculate fresh RDM lacking 3nY and without CAZ, and were grown for five hours at 37 °C while shaking. Aliquots of 25 μL from each line (corresponding to ~10^7^ cfu) were plated on LB-agar, with or without 1 mM 3nY, in triplicate. Plates were allowed 5 minutes to dry in a sterile biosafety cabinet, and ceftazidime E-test MIC strips (Biomerieux) were added to each plate. Plates were incubated 16 hours at 37 °C. MIC values are reported as the lowest concentration at which no bacterial growth occurred, reported with standard error of the mean^58–65^.

### GFP Assays

The codon for Tyr39 (TAT) in GFPmut2, under control of the *tacI* promoter with *Lac* operator, was changed to the amber codon (TAG), or ochre nonsense codon (TAA) using Gibson cloning with oligo DT10 paired with DT08 (for TAG codon) or DT09 (for TAA codon). Gibson reaction was electroporated into TOP10 *E*. *coli*, and plated on LB-agar supplemented with 50 μg mL^−1^ kanamycin (KAN). Sequences were verified using Sanger sequencing at the University of Texas Core Facility.

Glycerol stocks of the eighteen evolved clones and six progenitor clones were used to inoculate 2 mL of LB supplemented with 10 mM 3nY and 2 or 22 μg mL^−1^ ceftazidime (progenitor and evolved cells, respectively), and grown to saturation overnight. A 5 μL aliquot from each was used to inoculate 5 mL of LB, 10 mM 3nY, and 2 μg mL^−1^ ceftazidime, and grown at 37 °C for 3 hours, and then made electrocompetent with serial washes of 10% glycerol. Samples were transformed with GFP variants, recovered with LB media for one hour at 37 °C, then plated on LB-agar supplemented with 10 mM 3nY, 0.02% glucose, 50 μg mL^−1^ KAN, and 2 or 22 μg mL^−1^ CAZ, and incubated overnight at 37 °C.

Four samples from each plate were picked and grown overnight at 37 °C in 500 μL LB with 10 mM 3nY, 0.02% glucose, 50 μg mL^−1^ KAN, and 2 or 22 μg mL^−1^ CAZ. A 2 μL aliquot of each overnight culture was used to inoculate 1 mL LB media supplemented with 50 μg mL^−1^ KAN, and containing either 1 mM 3nY, 1 mM 3iY, or with no supplemented ncAA. Cultures were grown at 37 °C for 3 hours, then induced with 1 mM IPTG, and grown for another 5 hours at 37 °C. Final cultures were rinsed twice with iterative centrifugation at 2000*g* and PBS (50 mM phosphate, 300 mM NaCl, pH 8) at 4 °C. Samples were resuspended in 1 mL PBS and a 150 μL aliquot was used to measure relative fluorescence by measuring absorbance at 600 nm (OD600), and fluorescence using a 475 nm excitation and measuring emission at 525 nm using Tecan Infinite M200 pro microplate reader.

### Data availability

All data generated or analyzed during this study are available from the corresponding author on reasonable request. Sequence data is available as NCBI, BioProject ID PRJNA430697.

## Electronic supplementary material

Supplementaty Information

Dataset 1
