# Supplementary material for: Evolving Bacterial Fitness with an Expanded Genetic Code
Source: Sci Rep. 2018 Feb 19;8:3288. doi: 10.1038/s41598-018-21549-w (PMC5818497; doi:10.1038/s41598-018-21549-w)
Supplement: Supplementary file 1 — Supplementaty Information [file 41598_2018_21549_MOESM1_ESM.pdf]

Evolving Bacterial Fitness with an Expanded Genetic Code – Supplementary Information

Tack, Drew S., Cole, Austin C., Shroff, Raghav, Morrow, Barrett.R., Ellington, Andrew D.

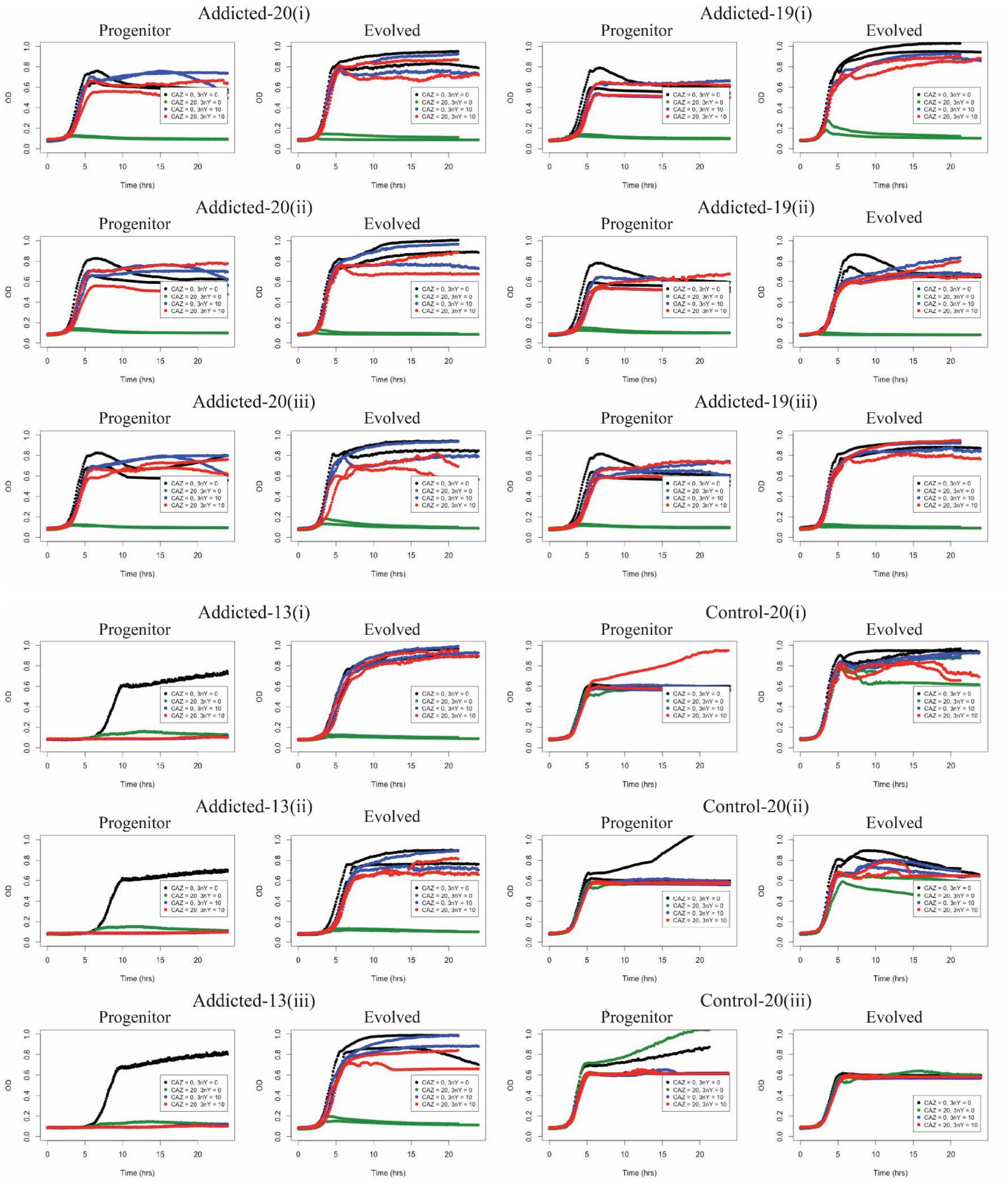

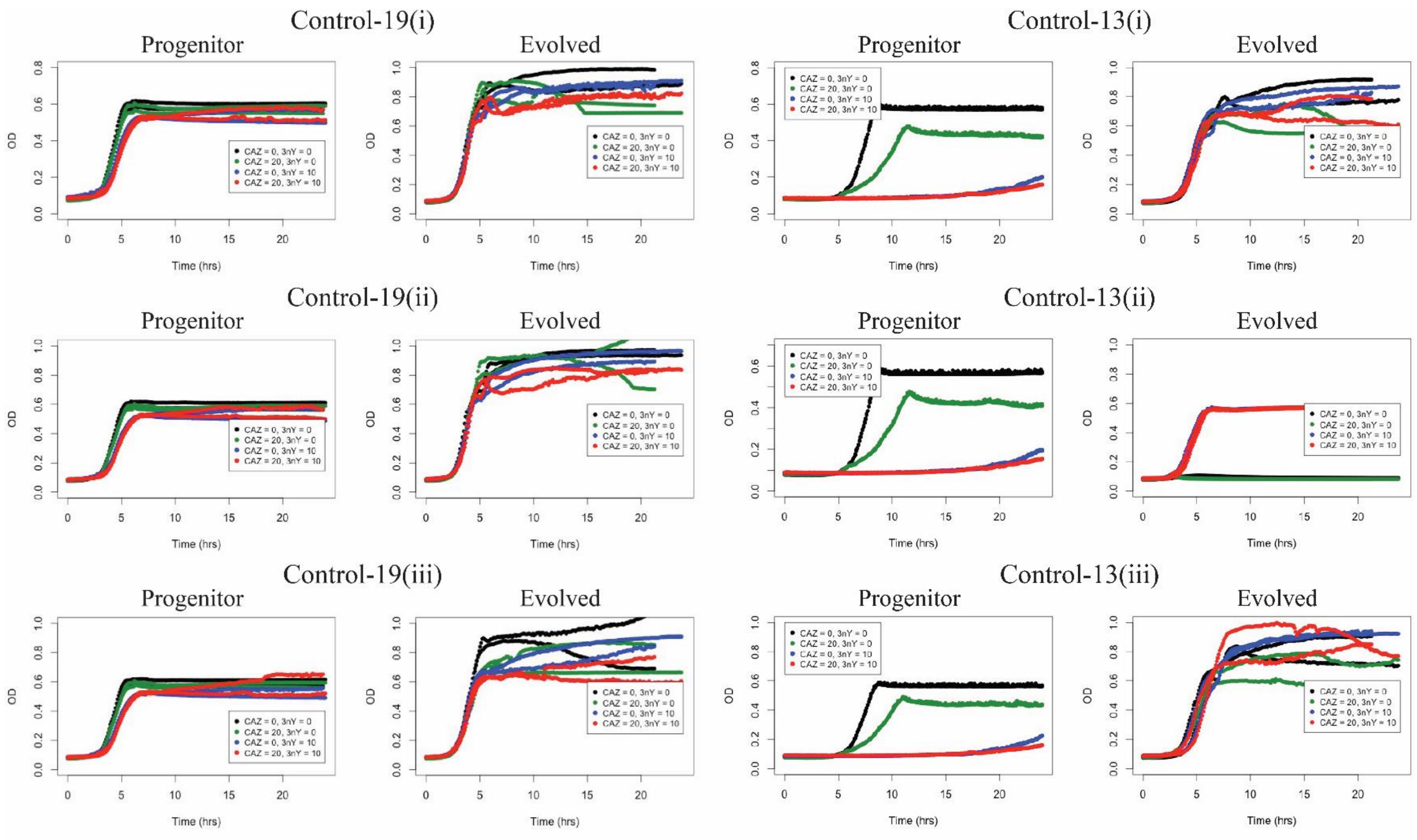

Supplementary Figure 1 – Growth curves of progenitor cells and evolved lines in RDM conditions. Progenitor cells transformed with pADDICTED or pCONTROL plasmid were initially incapable of growh in RDM-13 with 10 mM 3nY (green and red growth curves), but were fully capable after evolution. Notably, After 2000 generations of evolution, line Control-13(ii) was incapable of growth without 3nY, even in the absence of ceftazidime, due to an in-frame amber codon in essential gene *lptD*.

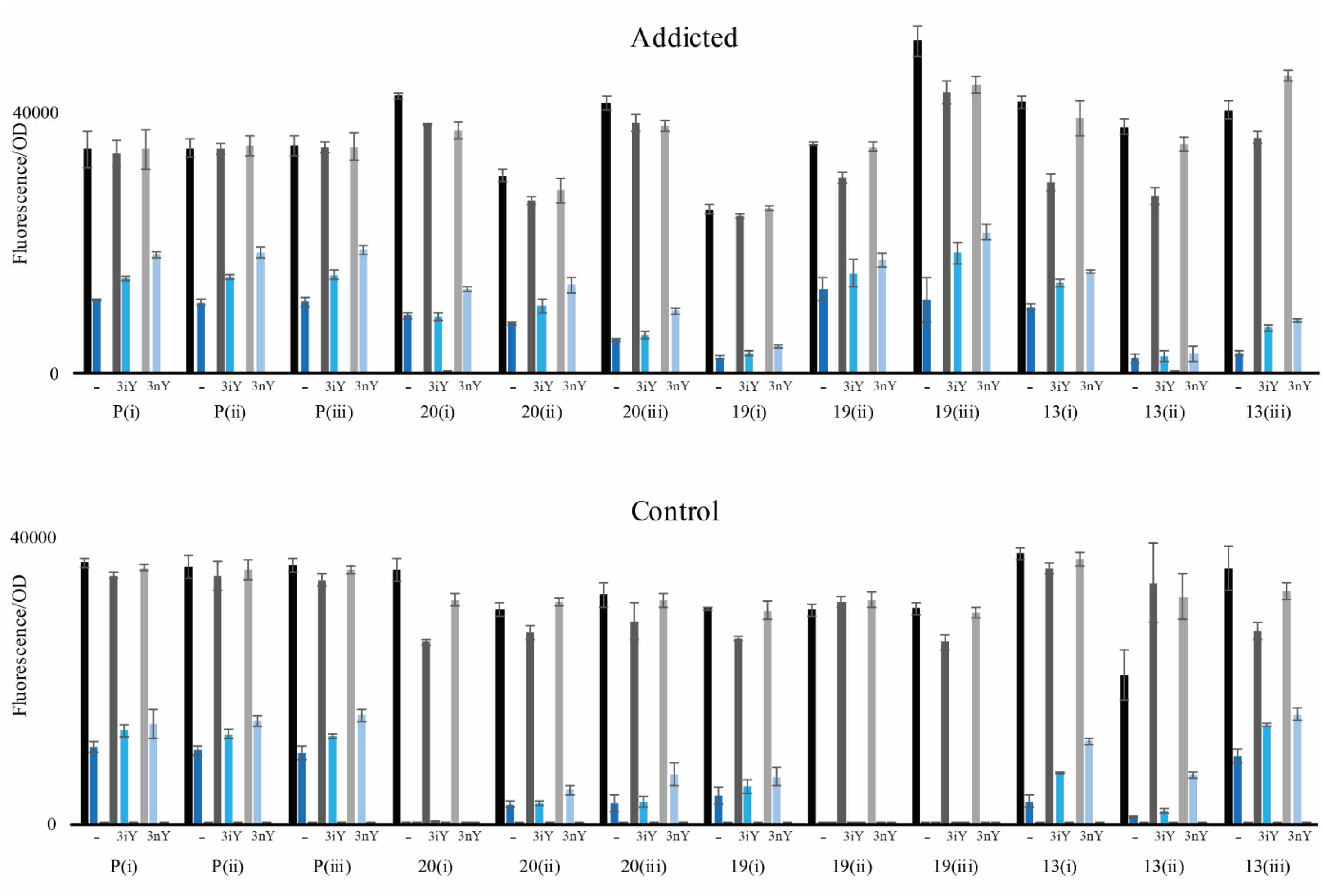

Supplementary Figure 2 – Expression levels of GFP variants in progenitor and evolved lines containing pADDICTED (top) and pCONTROL (bottom). Expression of all lines was tested in three media conditions: LB, LB with 1 mM 3nY, and LB with 1 mM 3iY. Three GFP variants were tested in each condition, with alternate codons at positions 39; 39-TAT (gray), 39-TAG (blue), and 39-TAA (orange, near zero).

RDM			RDM-20									RDM-19									RDM-13						
	ncAA		None			3iY			3nY			None			3iY			3nY			None			3iY			3nY
		Plasmid	none	pADD.	pCON.	none	pADD.	pCON.	none	pADD.	pCON.	none	pADD.	pCON.	none	pADD.	pCON.	none	pADD.	pCON.	none	pADD.	pCON.	none	pADD.	pCON.	ALL
RDM-20		none		0.446	0.385	0.002	0.323	0.529	0.000	0.520	0.341	0.007	0.478	1.000	0.000	0.000	0.000	0.000	0.000	0.099	0.000	0.000	0.005	0.000	0.000	0.000	0.000
		pADD.	0.446		0.552	0.277	0.120	0.499	0.143	0.089	0.056	0.220	0.089	0.284	0.057	0.001	0.004	0.001	0.000	0.003	0.033	0.000	0.000	0.005	0.000	0.000	0.000
	None	pCON.	0.385	0.552		0.071	0.047	0.599	0.013	0.113	0.029	0.058	0.090	0.402	0.001	0.000	0.000	0.000	0.000	0.002	0.000	0.000	0.000	0.000	0.000	0.000	0.000
		none	0.002	0.277	0.071		0.905	0.017	0.503	0.975	0.909	0.158	0.972	0.436	0.006	0.000	0.011	0.000	0.014	0.413	0.001	0.000	0.076	0.000	0.000	0.000	0.000
		pADD.	0.323	0.120	0.047	0.905		0.161	0.713	0.885	1.000	0.811	0.876	0.353	0.333	0.002	0.016	0.000	0.000	0.175	0.144	0.000	0.005	0.009	0.000	0.000	0.000
	3iY	pCON.	0.529	0.499	0.599	0.017	0.161		0.001	0.360	0.166	0.020	0.314	0.766	0.000	0.000	0.000	0.000	0.000	0.040	0.000	0.000	0.001	0.000	0.000	0.000	0.000
		none	0.000	0.143	0.013	0.503	0.713	0.001		0.938	0.724	0.027	0.932	0.249	0.051	0.000	0.005	0.000	0.004	0.371	0.002	0.000	0.038	0.000	0.000	0.000	0.000
		pADD.	0.520	0.089	0.113	0.975	0.885	0.360	0.938		0.856	0.803	1.000	0.353	0.703	0.099	0.189	0.062	0.001	0.208	0.513	0.000	0.006	0.199	0.002	0.037	0.000
	3nY	pCON.	0.341	0.056	0.029	0.909	1.000	0.166	0.724	0.856		0.819	0.849	0.281	0.353	0.002	0.014	0.000	0.000	0.092	0.158	0.000	0.001	0.011	0.000	0.000	0.000
RDM-19		none	0.007	0.220	0.058	0.158	0.811	0.020	0.027	0.803	0.819		0.782	0.490	0.000	0.000	0.000	0.000	0.001	0.195	0.000	0.000	0.011	0.000	0.000	0.000	0.000
		pADD.	0.478	0.089	0.090	0.972	0.876	0.314	0.932	1.000	0.849	0.782		0.332	0.673	0.071	0.151	0.040	0.001	0.213	0.470	0.000	0.007	0.156	0.001	0.022	0.000
	None	pCON.	1.000	0.284	0.402	0.436	0.353	0.766	0.249	0.353	0.281	0.490	0.332		0.081	0.000	0.002	0.000	0.000	0.018	0.032	0.000	0.000	0.002	0.000	0.000	0.000
		none	0.000	0.057	0.001	0.006	0.333	0.000	0.051	0.703	0.353	0.000	0.673	0.081		0.000	0.019	0.000	0.006	0.496	0.054	0.000	0.047	0.000	0.000	0.000	0.000
		pADD.	0.000	0.001	0.000	0.000	0.002	0.000	0.000	0.099	0.002	0.000	0.071	0.000	0.000		0.662	0.133	0.111	0.659	0.007	0.005	0.000	0.271	0.643	0.005	0.000
	3iY	pCON.	0.000	0.004	0.000	0.011	0.016	0.000	0.005	0.189	0.014	0.000	0.151	0.002	0.019	0.662		0.130	0.097	0.825	0.134	0.001	0.244	0.913	0.007	0.226	0.000
		none	0.000	0.001	0.000	0.000	0.000	0.000	0.000	0.062	0.000	0.000	0.040	0.000	0.000	0.133	0.130		0.355	0.429	0.000	0.005	0.547	0.001	0.042	0.748	0.000
		pADD.	0.000	0.000	0.000	0.014	0.000	0.000	0.004	0.001	0.000	0.001	0.001	0.000	0.006	0.111	0.097	0.355		0.046	0.021	0.010	0.909	0.125	0.554	0.226	0.000
	3nY	pCON.	0.099	0.003	0.002	0.413	0.175	0.040	0.371	0.208	0.092	0.195	0.213	0.018	0.496	0.659	0.825	0.429	0.046		0.711	0.000	0.120	0.802	0.043	0.409	0.000
RDM-13		none	0.000	0.033	0.000	0.001	0.144	0.000	0.002	0.513	0.158	0.000	0.470	0.032	0.054	0.007	0.134	0.000	0.021	0.711		0.000	0.101	0.001	0.000	0.000	0.000
		pADD.	0.000	0.000	0.000	0.000	0.000	0.000	0.000	0.000	0.000	0.000	0.000	0.000	0.000	0.005	0.001	0.005	0.010	0.000	0.000		0.025	0.001	0.119	0.001	0.000
	None	pCON.	0.005	0.000	0.000	0.076	0.005	0.001	0.038	0.006	0.001	0.011	0.007	0.000	0.047	0.000	0.244	0.547	0.909	0.120	0.101	0.025		0.288	0.587	0.419	0.000
		none	0.000	0.005	0.000	0.000	0.009	0.000	0.000	0.199	0.011	0.000	0.156	0.002	0.000	0.271	0.913	0.001	0.125	0.802	0.001	0.001	0.288		0.006	0.141	0.000
		pADD.	0.000	0.000	0.000	0.000	0.000	0.000	0.000	0.002	0.000	0.000	0.001	0.000	0.000	0.643	0.007	0.042	0.554	0.043	0.000	0.119	0.587	0.006		0.019	0.000
	3iY	pCON.	0.000	0.000	0.000	0.000	0.000	0.000	0.000	0.037	0.000	0.000	0.022	0.000	0.000	0.005	0.226	0.748	0.226	0.409	0.000	0.001	0.419	0.141	0.019		0.000
	3nY	ALL	0.000	0.000	0.000	0.000	0.000	0.000	0.000	0.000	0.000	0.000	0.000	0.000	0.000	0.000	0.000	0.000	0.000	0.000	0.000	0.000	0.000	0.000	0.000	0.000	

Supplementary Table 1 – Calculated p-values, from two-tailed t-tests, for comparisons of doubling times of wild-type MG1655 containing no plasmid (none), containing plasmid pADDICTED (pADD.), or transformed with plasmid pCONTROL (pCON.), and in RDM-20, -19, or 13, and with no ncAA, with 10 mM 3-iodo-tyrosine, or with 10mM 3-nitrotyrosine. p-values <.05 highlighted.

Oligo	Sequence
DT01	CCTAATGAGTGAACTCTTCCTTTTTCAATATTATTGAAGCATTTATCAGGG
DT02	CCCAGTCAGCTGTCAGACCAAGTTTACTCATATATACTTTAGATTGATTTCTG
DT03	GGTCTGACAGCTGACTGGGTTGAAGGCTCTCAAGG
DT04	TGAAAAAGGAAGAGTTCACTCATTAGGCACCCCAATCATCG
DT05	GATCGTTATTACGGCGAGTTGAATGAAGCCATACCAAACGACGAG
DT06	CAACTCGCCGTAATAACGATCCTAGCGAGTTACATGATCCCCCATGTTGTGC
DT07	CAACTCGCCGTAATAACGATCAAAGCGAGTTACATGATCCCCCATGTTGTGC
DT08	GGAGAGGGTGAAGGTGATGCAACATAGGGAAAACTTACCCTTAAATTTATTTGCACTACT
DT09	GGAGAGGGTGAAGGTGATGCAACATAAGGAAAACTTACCCTTAAATTTATTTGCACTACT
DT10	TGTTGCATCACCTTCACCCTCTCCACTGACAGAAAATTTGTGCCC

Supplementary Table 2 – Oligo sequences.
